# Combined impact of plasma phospho-tau 217, GFAP and NfL on longitudinal domain-specific cognitive decline

**DOI:** 10.1093/geroni/igaf122.3176

**Published:** 2025-12-31

**Authors:** Chao-Yi Wu, Liu Chen, Jennifer Gatchel, Sudeshna Das, Pia Kivisäkk, Steven Arnold, Hiroko Dodge

**Affiliations:** Massachusetts General Hospital, Harvard Medical School, Boston, Massachusetts, United States; Massachusetts General Hospital, Harvard Medical School, Boston, Massachusetts, United States; Massachusetts General Hospital, Boston, Massachusetts, United States; Massachusetts General Hospital, Harvard Medical School, Boston, Massachusetts, United States; Massachusetts General Hospital, Boston, Massachusetts, United States; Massachusetts General Hospital, Boston, Massachusetts, United States; Massachusetts General Hospital, Harvard Medical School, Boston, Massachusetts, United States

## Abstract

Clinical trials are increasingly focused on pre-manifest and early Alzheimer’s disease. Accurately predicting clinical progression is important to avoid unnecessary treatment and improve trial efficiency. Plasma p-tau217, an indicator of tau pathology with strong associations to amyloid-beta pathology, NfL, a marker of axonal damage and neurodegeneration and GFAP, a marker of inflammation, are promising diagnostic and prognostic tools. Their combined use could offer more accurate prognostic insights than either biomarker alone. We examined the trajectories of domain-specific cognitive functions by stratifying participants based on plasma p-tau217, NfL and GFAP levels (high/low). Participants were from the Massachusetts Alzheimer’s Disease Research Center cohort (n = 523). Cognitive functions were assessed using the National Alzheimer’s Coordinating Center Uniform Data Set v1-3: global cognition (CDR sum of box; MMSE, MoCA converted in v3), memory (Logical Memory), executive functions (Trail Making Test B), language (Boston Naming), and language-based executive function (Category Fluency Animals). We used linear mixed-effects models with 8 groups combining 3 plasma biomarkers to predict cognitive trajectories over 7 years, controlling for age, sex, and education. High p-tau217 alone was significantly associated with declines in Logical Memory (coefficient=-0.12; p = 0.03) and Boston Naming (coefficient=-0.16; p < 0.01), but not associated with decline in CDR sum of box and MMSE unless combined with a high burden of NfL and/or GFAP (CDR group*time coefficients=0.17-0.34, p < 0.01; MMSE group*time coefficients=-0.39 to -0.69, p < 0.01). Neither high GFAP alone nor high NfL alone was associated with significant cognitive declines. The combined use of plasma biomarkers provides a promising approach for predicting domain-specific cognitive decline.

